# Combined brain network topological metrics with machine learning algorithms to identify essential tremor

**DOI:** 10.3389/fnins.2022.1035153

**Published:** 2022-11-02

**Authors:** Qin Li, Li Tao, Pan Xiao, Honge Gui, Bintao Xu, Xueyan Zhang, Xiaoyu Zhang, Huiyue Chen, Hansheng Wang, Wanlin He, Fajin Lv, Oumei Cheng, Jing Luo, Yun Man, Zheng Xiao, Weidong Fang

**Affiliations:** ^1^Department of Radiology, The First Affiliated Hospital of Chongqing Medical University, Chongqing, China; ^2^Department of Neurology, The First Affiliated Hospital of Chongqing Medical University, Chongqing, China

**Keywords:** graph theory, multiple thresholds, machine learning, resting-state functional magnetic resonance imaging, essential tremor

## Abstract

**Background and objective:**

Essential tremor (ET) is a common movement syndrome, and the pathogenesis mechanisms, especially the brain network topological changes in ET are still unclear. The combination of graph theory (GT) analysis with machine learning (ML) algorithms provides a promising way to identify ET from healthy controls (HCs) at the individual level, and further help to reveal the topological pathogenesis in ET.

**Methods:**

Resting-state functional magnetic resonance imaging (fMRI) data were obtained from 101 ET and 105 HCs. The topological properties were analyzed by using GT analysis, and the topological metrics under every single threshold and the area under the curve (AUC) of all thresholds were used as features. Then a Mann-Whitney *U*-test and least absolute shrinkage and selection operator (LASSO) were conducted to feature dimensionality reduction. Four ML algorithms were adopted to identify ET from HCs. The mean accuracy, mean balanced accuracy, mean sensitivity, mean specificity, and mean AUC were used to evaluate the classification performance. In addition, correlation analysis was carried out between selected topological features and clinical tremor characteristics.

**Results:**

All classifiers achieved good classification performance. The mean accuracy of Support vector machine (SVM), logistic regression (LR), random forest (RF), and naïve bayes (NB) was 84.65, 85.03, 84.85, and 76.31%, respectively. LR classifier achieved the best classification performance with 85.03% mean accuracy, 83.97% sensitivity, and an AUC of 0.924. Correlation analysis results showed that 2 topological features negatively and 1 positively correlated with tremor severity.

**Conclusion:**

These results demonstrated that combining topological metrics with ML algorithms could not only achieve high classification accuracy for discrimination ET from HCs but also help us to reveal the potential topological pathogenesis of ET.

## Introduction

The 2018 Movement Disorders Consensus Criteria redefined essential tremor (ET) as a common movement syndrome characterized by isolated bilateral upper limb action tremor for at least 3 years and without other neurological signs (Bhatia et al., 2018). It is reported that more than 60 million people worldwide are affected, and the incidence increases with age (Welton et al., 2021). Although a large number of researches has revealed that the abnormalities in the cerebello-thalamo-cortical networks were closely associated with tremor in ET (Nicoletti et al., 2020), its pathogenesis mechanism, especially brain network topological properties changes, is still not well understood, and the lack of specific diagnostic markers also makes the diagnosis of ET difficult.

Resting-state functional magnetic resonance imaging (Rs-fMRI) has been widely used to study the brain network pathogenesis mechanisms of ET due to its good temporal and spatial resolution and high safety. Compared with the other common brain network analysis methods (i.e., regional homogeneity, degree centrality, and functional connectivity), the graph theory (GT) approach has been used to characterize the brain complex network topological properties in neurological diseases with the advantage of fully describing the topological properties of brain networks (Dai et al., 2019; De Micco et al., 2021; Suo et al., 2021). Several studies have used the GT method and have found that loss of small-world characteristics and the alterations of degree centrality, nodal local efficiency, and nodal betweenness centrality in motor and no-motor areas were related to ET patients (Jang et al., 2016; Benito-León et al., 2019b; Li et al., 2021; Novaes et al., 2021; Yang et al., 2021). However, the methods of these studies were traditional mass univariate analyses, and they could not be used to diagnose individual ET patients, and the topological properties based on network sparsity thresholds selection approaches have not been adopted.

Fortunately, these shortcomings can be remedied by machine learning (ML) approaches. ML builds the optimal models by learning and training from massive input data and then applies the model to new data to predict and analyze diseases based on a single-subject level (Myszczynska et al., 2020). In the past several years, the application of the ML method on ET research has been widely discussed and achieved relatively good results (Benito-León et al., 2019a; Prasad et al., 2019; Suppa et al., 2021). However, up to now, no study has combined the brain network topological properties based on GT analysis with ML to identify ET patients.

Therefore, considering the potential advantages of ML, we explored whether combining the brain network topological metrics based on GT analysis with multiple ML algorithms could be used to identify ET patients from healthy controls (HCs). It is worth noting that the brain network topological properties metrics under every single threshold and the area under the curve (AUC) values for each topological metric over the sparsity range were all selected as features for model building in our study. We hypothesized that these models could achieve good classification performance and these significant discriminative features could help to reveal the changes of the underlying brain network topological properties in ET.

## Materials and methods

### Participants

ET patients were recruited at the movement disorders outpatient clinic of the First Affiliated Hospital of Chongqing Medical University. HCs were recruited from the local area through poster advertisements and evaluated by experienced neurologists. The inclusion criteria for subjects were as follows: (1) the diagnosis of ET patients met the 2018 Movement Disorders Consensus Criteria (Bhatia et al., 2018), and all ET patients had annual follow-ups through the outpatient department or by telephone; (2) the tremor onset age of patients is between 18 and 55 years old, and patients with earlier or later tremor onset age were not included; (3) the patients were without any apparent cognitive impairment [Mini-Mental State Examination (MMSE) scores > 24] and were right-handed; (4) the patients presented with moderate or greater amplitude kinetic tremor (tremor rating ≥ 2 during at least three tests); (5) the ET patients were without PD, parkinsonism, dystonia, ataxia, thyroid disease, stroke, epilepsy, brain injury, or any other neurological dysfunction; (6) all participants had no evidence of vascular or structural brain defects on T2- or T1-weighted images; (7) all participants met the image quality and head motion control criteria (see Supplementary Text 1). After controlling image quality and head motion, 2 ET patients and 3 HCs with arachnoid cysts and 2 ET patients and 4 HCs with FD_power head motion > 0.2 mm were removed from our study. Finally, 101 ET and 105 age- and sex-matched HCs were included in our study. In addition, none of the HCs reported having a first-degree or second-degree relative with ET or PD. Each subject signed an informed consent form approved by the ethics committee of the First Affiliated Hospital of Chongqing Medical University (Chongqing, China), and the study was performed in accordance with the Declaration of Helsinki of the World Medical Association.

Tremor severity was assessed with the Fahn-Tolosa-Marin Tremor Rating Scale (FTM-TRS) (Fahn et al., 1988) and the Essential Tremor Rating Assessment Scale (TETRAS) (Elble et al., 2012). The depression severity of each patient was assessed by the Hamilton Depression Rating Scale (HDRS-17) (Bobo et al., 2016), and the anxiety severity of each patient was assessed by the Hamilton Anxiety Rating Scale (HARS-14) (Bruss et al., 1994). The MMSE was used to briefly evaluate cognitive function and to screen for dementia.

### Data acquisition

All subjects were scanned on GE Signa Hdxt 3-T scanner (General Electric Medical Systems, Milwaukee, WI, USA) equipped with a standard 8-channel head coil at the First Affiliated Hospital of Chongqing Medical University. Foam padding and rubber earplugs were used to control head motion and attenuate scanner noise. During Rs-fMRI scanning, all subjects were required to relax, think of nothing, keep their eyes closed but not fall asleep, and move as little as possible. Rs-fMRI images were collected using an echo-planar imaging (EPI) pulse sequence with the following parameters: 33 axial slices, repetition time (TR) = 2,000 ms, echo time (TE) = 40 ms, flip angle = 90°, slice thickness = 4.0 mm, slice gap = 0 mm, acquisition order = interleaved (interleaved scans and the slice order is 1:2:33, 2:2:32, and the reference slice is 33), field of view (FOV) = 240 × 240 mm, matrix size = 64 × 64, and a total of 240 volumes were obtained (duration = 8 min). High-resolution 3D T1-weighted images (TR = 8.3 ms, TE = 3.3 ms, flip angle = 15°, slice thickness/gap = 1.0/0 mm, FOV = 240 × 240 mm, and matrix = 256 × 192) and T2-weighted FLAIR images (TR = 8,000 ms, TE = 126 ms, TI = 1,500 ms, slice thickness/gap = 5.0/1.5 mm, FOV = 240 × 240 mm, and matrix = 256 × 192) were also obtained. We did not use the T2-weighted FLAIR images for data processing, but they were used for image evaluation and data quality assessment.

### Data preprocessing

Data preprocessing was performed using the DPARSFA toolbox version 2.2^1^ on MATLAB (MathWorks Inc., Natick, MA, USA) platform, which involved: (1) Removal of the first 10 time points for signal stabilization. (2) Slice timing correction. (3) Realignment. This step realigns individual images such that each part of the brain in every volume is in the same position. (4) T1 segmentation and spatial normalization. The 3D-T1 images were co-registered to the mean Rs-fMRI data for each subject. Specifically, 3D T1-weighted images were divided into white matter (WM), gray matter (GM), and cerebrospinal fluid (CSF) probability maps using SPM DARTEL segmentation, and CSF images were resampled to 1.5-mm isotropic voxels, spatially normalized to the MNI space using affine transformation and non-linear deformation, and then, resampled to 3-mm isotropic voxels resolution with Rs-fMRI and the deformation field was applied to the Rs-fMRI data. (5) Spatial smoothing with a 4-mm full-width at half-maximum Gaussian kernel. (6) Regressing out nuisance covariates: global, WM, CSF signals, and Friston 24 head motion parameters. (7) Detrending and filtering. Linear detrending and temporal band-pass filtering at 0.01–0.08 Hz was used to remove low-frequency drift and high-frequency physiological noise.

### Network construction

The functional brain network is composed of nodes and edges between nodes. To better study the alterations of the brain network topological properties in ET patients, a functional segmentation atlas, the Dosenbach atlas was adopted (Dosenbach et al., 2010), and it divides the brain into 160 nodes, including six networks, namely the CON, frontal-parietal, default, sensorimotor, occipital, and cerebellum. Extracting the average time series of each node, and the edges were defined as the Pearson correlation coefficients of the average time series between each pair of nodes. And then, the raw 160 × 160 brain connectivity (BC) matrix was obtained. To improve the normality, the BC matrix was transformed into a Z-score matrix by using Fisher’s r-to-z transformation. Finally, the Z-score matrix was converted to a binary adjacency matrix based on a wide range of sparsity thresholds (S) (as defined below).

### Network topological properties metrics

Further network analysis was based on the binary adjacent matrices at a wide range of sparsity thresholds (S). S is defined as the ratio of the number of existing edges divided by the maximum possible number of edges in the network. There is no unified rule for the selection of network sparsity. Therefore, a wide range of S was used to ensure that the threshold network was evaluable for the small-world and to minimize the number of spurious edges in each network (Zhang et al., 2011). The threshold range of our study was 0.05–0.50 with an interval of 0.05 (a total of 10 thresholds were defined).

The global and nodal topological metrics were analyzed by GRETNA software (Wang et al., 2015) on MATLAB (MathWorks Inc., Natick, MA, USA) platform. The global topological metrics include (1) small-world measures: the clustering coefficient (Cp), characteristic path length (Lp), normalized clustering coefficient (γ), normalized characteristic path length (λ), and small-worldness (σ); (2) network efficiency measures: global efficiency (Eglobal) and local efficiency (Elocal). The nodal topological properties include the nodal clustering coefficient (NCp), nodal efficiency (Ne), nodal local efficiency (NLe), degree centrality (DC), and nodal betweenness centrality (BC). These brain network topological properties are defined and explained in the study by Rubinov and Sporns (2010). The brain network global and nodal topological properties on the 160 ROIs under 10 thresholds were selected as features.

Moreover, for each network metric, the AUC was calculated over 0.05 ≤ S ≤ 0.50 with an interval of 0.05. The AUC provides a summarized scalar that does not rely on specific threshold selection and is a sensitive way to detect topological alterations in the brain network (Zhang et al., 2011). The AUC values of each topological metric on the 160 ROIs were also selected as features.

### Feature selection

As mentioned above, there were a total of 8,877 features for each participant. However, a large number of original features in the dataset will easily fall into a “curse of dimensionality,” which slows down the running speed of the algorithm and reduces the accuracy of the model. By identifying the key features of the data, feature selection can improve the learning efficiency of ML models and reduce the probability of overfitting (Remeseiro and Bolon-Canedo, 2019).

In our study, firstly, the data was divided into a 70% training set and a 30% testing set by stratified sampling. Next, we combined the Mann-Whitney *U*-test and LASSO as the feature selection method for the 8,877 features. Specifically, features with *P* < 0.01 were retained after the Mann-Whitney *U*-test. Secondly, Spearman’s rank correlation analysis was used to eliminate multicollinearity between any two feature columns. If the correlation coefficient of the two features was greater than 0.7, the latter one was removed. Thirdly, data standardization was conducted for the features selected by Spearman’s rank correlation. Finally, by constructing a lost function, LASSO (L1 regularization) resets the weight of unimportant features to 0 to achieve the purpose of accurate prediction with fewer features (Tibshirani, 1996). The lost function of LASSO is as follows:

$$\text{L}=\sum_{i=1}^{n}{\left(y_{i}-{\hat{y}}_{i}\right)}^{2}+\lambda \,\sum_{j=1}^{p}\left|\beta_{j}\right|$$

where $\lambda \,\sum_{j=1}^{p}\left|\beta_{j}\right|$ is the L1 regularization penalty on the coefficient β_*j*_ and λ is the shrinkage parameter that needs to be determined before performing the learning task (Ueno et al., 2021). Therefore, LASSO with fivefold cross-validation was used to further select important features by finding the optimal λ and calculate the weight coefficients of each feature.

### Model building and evaluation

After selecting the discriminative features of the brain functional network, four classifiers, including support vector machine (SVM), logistic regression (LR), random forest (RF), and Gaussian Naïve Bayes (GaussianNB) algorithms were used to construct models for classification (all steps of feature selection and model construction were just performed in the training set). The applications of these classifiers are as follows:

As a supervised learning model, SVM can automatically learn the classification hyperplane in feature space by finding the maximum boundary distance (Hsu et al., 2003), and the performance of classification highly depends on the selection of hyperparameters. Therefore, in our study, grid-search strategy with 10-fold cross-validation (GS-10 CV) was applied to find the optimal hyperparameters (the penalty parameter C and kernel width parameter γ) and kernel function. It means that we will apply the combination of parameters with the highest accuracy in the GS-10 CV to the testing set.

RF is ensemble learning by constructing a set (ensemble) of trained decision trees (base estimators) to predict the outcome variable (Badillo et al., 2020). It works based on a consensus among decision trees. In most cases, ensemble learning can obtain better classification results than a single learner. Additionally, the RF can effectively avoid overfitting (Breiman, 2001) and require less parameter tuning (Segal, 2004). In our study, we performed parameter optimization for the base estimators.

LR is a probability statistical classification model, which uses the probability score as the predicted value of the dependent variable to measure the relationship between a classification dependent variable and one or more independent variables (Zhang et al., 2015). In our study, L1-regularized LR was used to avoid overfitting and improve the performance of the classification model, and Cs was used for parameter optimization.

The Naïve Bayes classifier is based on calculating simple statistics from a given training data set as a learning step, following the conditional probability of the Bayesian formula for classification (Badillo et al., 2020). In Bayes’ formula, assuming that conditions are independent between features, calculate the conditional probability of Y (classify into certain categories) in the case of X (feature distribution) occurrence. In our study, the feature distribution was assumed to be Gaussian, and Gaussian Naïve Bayes (GaussionNB) was used for classification. The Bayes’ formula is as follows:

$$P\left(Y|X\right)=\frac{P\,{\left(X|Y\right)}^{*}\,P\,\left(Y\right)}{P\,\left(X\right)}$$

**FIGURE 1:**
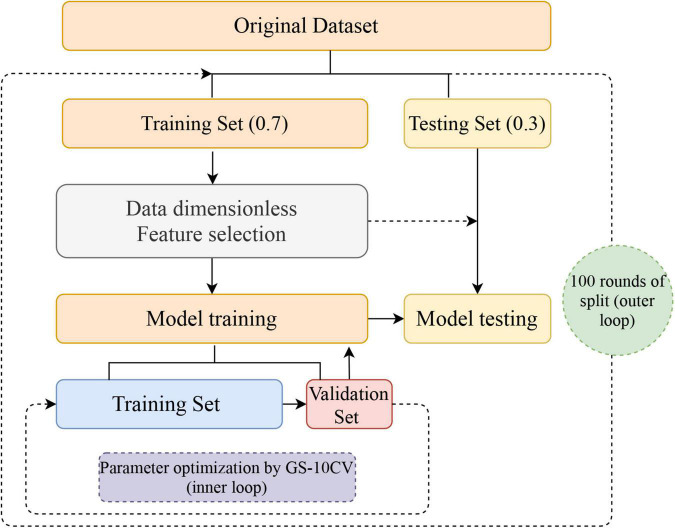
Flowchart for building a classification model. After 100 nested loops, the mean values of the accuracy, balanced accuracy, the area under the receiver operating characteristic curve, sensitivity, and specificity of the models were generated. GS-10CV, grid-search strategy with 10-fold cross-validation.

Furthermore, considering the instability and sampling bias due to a single split of the dataset, we repeated the above process 100 rounds (nested loop) to obtain 100 accuracy, sensitivity, specificity, and AUC values, which are shown as means ± standard deviations. The flow of the nested loop is shown in Figure 1. The performance of classification of each classifier was evaluated by mean accuracy, mean sensitivity, mean specificity, mean balanced accuracy, and mean AUCs. The corresponding formula is as follows:

$$A\,c\,c\,u\,r\,a\,c\,y=\frac{T\,P+T\,N}{T\,P+T\,N+F\,P+F\,N}$$

$$S\,e\,n\,s\,i\,t\,i\,v\,i\,t\,y=\frac{T\,P}{T\,P+F\,N}$$

$$S\,p\,e\,c\,i\,t\,i\,v\,i\,t\,y=\frac{T\,N}{T\,N+F\,P}$$

$$B\,a\,l\,a\,n\,c\,e\,d\,a\,c\,c\,u\,r\,a\,c\,y=\frac{S\,e\,n\,s\,i\,t\,i\,v\,i\,t\,y+S\,p\,e\,c\,i\,t\,i\,v\,i\,t\,y}{2}$$

TP, TN, FP, and FN refer to true positive, true negative, false positive, and false negative values, respectively. Accuracy refers to the proportion of all samples that are correctly identified. Sensitivity is also known as the true positive rate (TPR), which is the proportion of all truly positive samples identified as positive. Specificity is the proportion of all truly negative samples identified as negative. Balanced accuracy was defined as the average of sensitivity and specificity in each group. The AUC value is the area under the ROC curve and the coordinate axis. The closer the area is to 1, the stronger the recognition ability is. To test the significance of model performance, the permutation test was used to assess whether the accuracy and AUC values were significantly higher than chance (Shi et al., 2022a). Specifically, the class labels (1:ET, 0:HCs) of the data were shuffled 1,000 times and then conducted the above classification procedure to obtain the permutated accuracy and AUCs. The *p*-value was calculated by the probability of permutated accuracy and AUCs greater than the actual accuracy and AUCs. If the *p*-value was less than 0.05, it could be demonstrated that the classifier has a reliable classification performance.

### Statistical analyses

The demographics and clinical characteristics statistical processing and analyses were performed with SPSS. All ML analyses were based on scikit-learn in Python 3.8.8. For demographics and clinical characteristics, the Kolmogorov-Smirnov test was used to assess the normality of continuous variables, continuous and normally distributed variables were analyzed with two-sample *t*-tests, non-normally distributed variables were analyzed with the Mann-Whitney *U*-test, and sex was tested by chi-squared test, *P* < 0.05 demonstrated a significant difference. Finally, a Partial Pearson correlation was conducted to detect relationships between the selected features and the clinical tremor characteristics in the ET group with Bonferroni multiple comparison corrections (age, gender, education, head-motion parameters, and the scores on HDRS-17, HARS-14, and MMSE as covariates).

## Results

### Demographics and clinical characteristics

Demographic and clinical data of all participants are summarized in Table 1. The ET group had a lower score on the MMSE than the HCs group (*P* = 0.0003). There were no significant differences in age, gender, education, handedness, cigarette smoking, and the score on HDRS-17 and HDRS-14 between ET and HCs groups (*P* > 0.05). In addition, there was no significant difference in scrubbing volumes (*P* = 0.2437) and the mean FD estimates of power (*P* = 0.5562) between the two groups.

**TABLE 1:** Demographic and clinical features of ET and HCs.

Measure	ET	HCs	Statistics	*P*-value
**Demographic**				
Sample size	101	105	NA	NA
Age (years)	46.49 ± 14.75	45.03 ± 13.14	*T* = 0.75	0.4547
Gender (M:F)	48:53	61:44	*Z* = −1.51	0.1296
Education (years)	12.45 ± 4.33	12.59 ± 4.76	*T* = 0.23	0.8197
Handedness (R/L)	101:0	105:0	*Z* = 0.00	1.0000
Cigarette smoking	37/64	30/75	*Z* = −1.23	0.2180
**Clinical of tremor**				
Tremor of onset (years)	34.76 ± 11.01	NA	NA	NA
Tremor of duration (years)	11.72 ± 8.42	NA	NA	NA
Positive family history		NA	NA	NA
Positive	34	NA	NA	NA
Negative	67	NA	NA	NA
Alcohol sensitivity		NA	NA	NA
Positive	40	NA	NA	NA
Negative	51	NA	NA	NA
NA	10	NA	NA	NA
Tremor medication		NA	NA	NA
Propranolol	27 (40.93 ± 19.07 mg)	NA	NA	NA
Tremor symmetry		NA	NA	NA
R = L	81	NA	NA	NA
R < L	7	NA	NA	NA
R > L	13	NA	NA	NA
Tremor frequency	6.86 ± 2.55	NA	NA	NA
TRS-parts A&B	23.60 ± 8.53	NA	NA	NA
TRS-part C	12.81 ± 7.89	NA	NA	NA
TETRAS	21.16 ± 7.28	NA	NA	NA
TET-ADSL	12.69 ± 6.08	NA	NA	NA
**Clinical of psychology and cognitive**				
HDRS-17	2.06 ± 1.09	2.17 ± 1.40	*T* = −0.64	0.5234
HARS-14	2.69 ± 1.00	2.66 ± 1.86	*T* = 0.17	0.9640
MMSE	28.44 ± 1.36	29.10 ± 1.20	*T* = −3.69	0.0003
**Head movement**				
FD_power	0.10 ± 0.06	0.01 ± 0.06	*T* = 0.59	0.5562
Scrubbing volumes	15.21 ± 7.95	16.61 ± 9.12	*T* = −1.17	0.2437

ET, essential tremor; HCs, healthy controls; HDRS-17, 17-item Hamilton Depression Rating Scale; MMSE, Mini-Mental State Examination; HARS-14, 14-item Hamilton Anxiety Rating Scale; TRS, Fahn-Tolosa-Marin Tremor Rating Scale.

### The significant discriminative features

**FIGURE 2:**
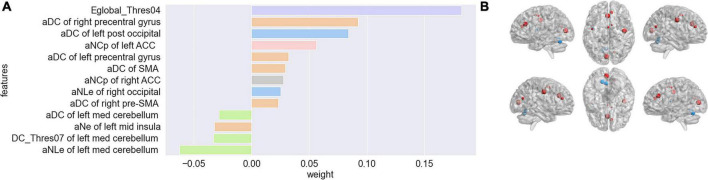
The top 13 selected features were obtained after the Mann-Whitney *U*-test and LASSO and the corresponding feature weight (feature weight was arranged from positive to negative) **(A)**. A represents a threshold-independent selection. NLe, nodal local efficiency; DC, degree centrality; NCp, nodal clustering coefficient; Ne, nodal efficiency. Five different colors represent different subnetworks (green-cerebellum, blue-occipital, pink-cingulo-opercular, orange-sensorimotor, gray-DMN). The brain regions correspond to the 13 features selected by the Mann-Whitney *U*-test and LASSO and the alterations of topological metrics **(B)**. Red represents a significantly increased topological metric value in ET compared with HCs and blue represents a significantly decreased topological metric value in ET compared with HCs (*P* < 0.01). The larger the ball, the higher the frequency.

During the given range of sparsity thresholds, both participants with ET and HCs showed small-world properties in the functional brain network with γ > 1 and λ≈1. Firstly, after feature screening by the Mann-Whitney *U*-test and LASSO, features with a frequency of more than 80 times in the 100 outer loops were regarded as features with a significant discriminative ability to distinguish ET from HCs, a total of 13 features. The brain region information, frequency, and feature weight of the 13 features were presented in Figure 2A and Supplementary Table 1, the feature with the highest frequency was the degree centrality located in the left post occipital, and the feature with the highest weight was the global efficiency with a 0.20 threshold and a weight of 0.1825. The other significant discriminative features were mainly located in the cerebellum, sensorimotor, occipital, cingulo-opercular (CON), and default mode network (DMN). Specifically, as shown in Supplementary Table 2, patients with ET were characterized by decreased degree centrality and nodal local efficiency in the cerebellum, increased degree centrality and nodal local efficiency in the sensorimotor network and occipital, increased nodal clustering coefficient in the bilateral ACC, and decreased nodal efficiency in the left middle insula. Meanwhile, the significant features include the global efficiency with a 0.20 threshold, which has a higher value in ET than in HCs. Figure 2B shows the increase or decrease of topological measures in different brain regions in ET compared with HCs.

### Classification

**FIGURE 3:**
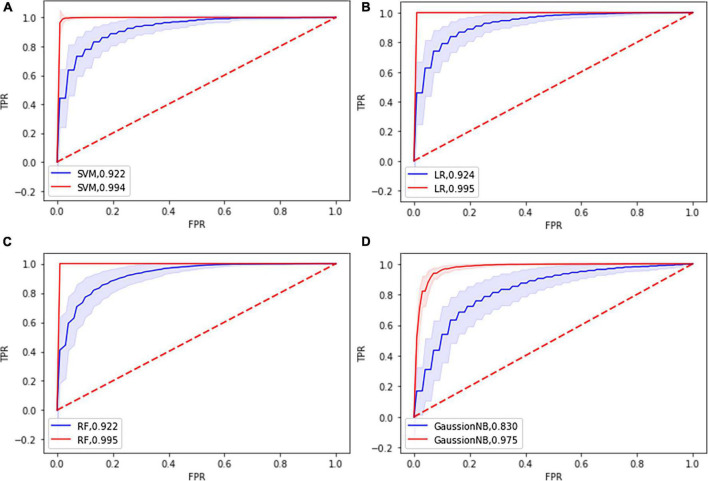
Receiver operating characteristic curve (ROC) of the four classifier models based on the topological features in the training dataset (blue) and in the testing dataset (red). **(A)** SVM: support vector machine; **(B)** LR: Logistic Regression; **(C)** RF: Random Forest; **(D)** GuassionNB: gaussian naïve bayes.

The classification performance of each classifier is shown in Table 2 and Supplementary Table 3. The LR classifier achieved the best classification performance with 85.03% mean accuracy and 85.00% mean balanced accuracy and 83.97% sensitivity and a mean AUC of 0.924 among the four ML algorithms. The RF achieved the classification performance with 84.85% mean accuracy and 84.85% mean balanced accuracy and 84.73% mean sensitivity and a mean AUC of 0.922. The SVM achieved the classification performance with 84.65% mean accuracy and 84.63% mean balanced accuracy and 84.03% mean sensitivity and a mean AUC of 0.922. The GaussionNB achieved the classification performance with 76.31% mean accuracy and 76.28% mean balanced accuracy and 75.57% mean sensitivity and a mean AUC of 0.830. The results of the permutation test showed that the accuracy and AUC values were significantly higher than chance (*P* < 0.001 for all loops). Figure 3 shows the ROC curves in the training and testing set for the four classifiers.

**TABLE 2:** Classification performance of multiple classifiers in the testing dataset.

Model	Testing dataset				
	mACC (%)	mb-ACC (%)	mSEN (%)	mSPE (%)	mAUC
SVM	84.65 ± 4.83	84.63 ± 4.85	84.03 ± 7.65	85.22 ± 6.40	0.922
LR	85.03 ± 4.49	85.00 ± 4.49	83.97 ± 6.87	86.03 ± 6.69	0.924
RF	84.85 ± 4.98	84.85 ± 4.98	84.73 ± 6.79	84.97 ± 6.71	0.922
GaussionNB	76.31 ± 6.21	76.28 ± 6.21	75.57 ± 8.78	77.00 ± 8.81	0.830

Data are shown as means ± standard deviations. mACC, mean accuracy; mb-ACC, mean balanced accuracy; mSEN, mean sensitivity; mSPE, mean specificity; mAUC, mean area under receive operator curve. SVM, support vector machine; RF, random forest; LR, logistic regression; GussianNB, guassian naïve bayes.

### Correlation between selected 13 features and clinical tremor characteristics

**FIGURE 4:**
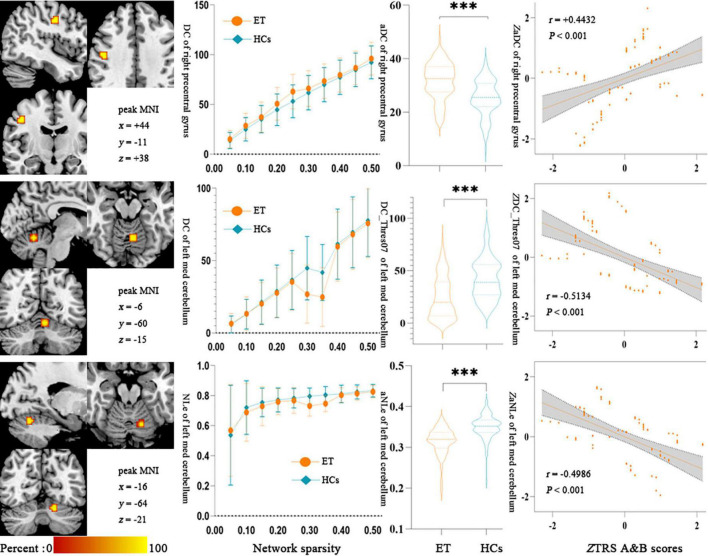
Results of Partial Pearson correlation analysis between the topologic metrics values of significant discriminative features and the TRS A&B scores in ET patients. Bonferroni multiple comparison corrections, corrected *P* < 0.05/20*(20–1)/2. **(Left)** The cluster of the significant discriminative features. **(Middle)** Mean and standard deviations of the significant discriminative features across subject groups (orange-ET, green-HCs) as a function of network sparsity and the mean topologic metric values of significant discriminative features with group differences across the sparsity from 0.05 to 0.50 between ET patients and HCs. ***P < 0.001. **(Right)** Scatterplots for the correlation analysis in patients with ET. NLe, nodal local efficiency; DC, degree centrality; zNLe, z-transformed nodal local efficiency; zDC, z-transformed nodal degree centrality; Thres07, the threshold of 0.35; aDC, the AUC values of degree centrality; zTRS A&B scores, z-transformed Fahn-Tolosa-Marin Tremor Rating Scale parts A and B scores; ET, essential tremor; HCs, healthy controls.

The Partial Pearson correlation analysis revealed that 3 discriminative features were significantly associated with the clinical tremor aspects, and Figure 4 shows the results of the correlation analysis. The degree centrality with a 0.35 threshold in the left med cerebellum and nodal local efficiency in the left med cerebellum were negatively correlated with the TRS parts A&B scores (*P* < 0.001, *r* = −0.5134 and −0.4986, respectively), and the degree centrality in the right precentral gyrus was positively correlated with the TRS parts A&B scores (*P* < 0.001, *r* = +0.4432). There was no significant correlation between other topological features and other clinical tremor characteristics in ET patients.

## Discussion

In this study, we first combined multiple ML algorithms with brain network topological metrics to identify ET from HCs, and three main results were gained. First, all of the four ML algorithms could achieve good classification performance, and the LR algorithm is the best. Second, the 13 topological metrics (12 nodal and 1 global) acted as significant discrimination features and were mainly located in cerebellar-motor and non-motor cortical networks. Third, some brain network topological metrics could be used to explain partially tremor clinical features.

In our study, four common classifiers were used to construct models and obtained excellent results in both training and testing sets, especially the model constructed by LR (penalty = L1) was considered the best model with the highest mean AUC of 0.924 in the testing set. The LR classifier is a statistical modeling technique that estimates the probability of a dependent variable relating to a set of independent variables through the sigmoid function (Ya et al., 2022), which has the advantages of simple implementation, good model interpretability, high numerical stability, and it is not easy to overfit. Among them, the penalty = L1, also called Lasso regularization, plays a key role by resetting the non-significant feature coefficients to zero. Meanwhile, other models also achieved mean AUCs over 0.8 with different advantages. For example, SVM incorporates several advantageous properties to reduce overfitting and deliver good generalization performance despite a small sample size (Mo et al., 2019; Shi et al., 2022b), RF has strong adaptability to the data on account of ensemble strategy and the prediction accuracy is relatively accurate (Chong-Wen et al., 2022), and NB performs well on small-scale data and has stable classification efficiency. However, this is only a preliminary and small-scale study, and the generalizability of the model across different groups, cultures, and ethnicities needs to be further verified before our model can be applied to clinical work.

Although the underlying physiological mechanism reflected by GT analysis is not very clear, most researchers accepted that the GT analysis has a great advantage in comprehensively characterizing the topological properties of the brain complex network (Benito-León et al., 2019b; Xu et al., 2020; Li et al., 2021), and these merits are not only confined to a lot of metrics such as global and nodal topological properties but also could be used to explore brain network pathogenesis mechanisms in neurological and neurodegenerative diseases. Consistent with the above studies, our results revealed that these brain network topological metrics can serve as significant discrimination features to identify ET from HCs, and the correlation analysis showed that some brain network topological metrics could be used to explain partially clinical tremor features. Previous studies revealed that the nodal and global topological metrics such as the changes in small-world or global efficiency are associated with ET patients, and our results showed that the nodal topological metrics and Eglobal with a 0.20 threshold acted as the significant discrimination features to identify ET from HCs. These aspects seemed to suggest our results were consistent with previous studies. However, it is worth noting that our results outperform existing research on GT combined with ML on ET. The results may have several possible explanations. First, the etiological, clinical, pathological, and therapeutic heterogeneity of ET may cause a variety of results among different research groups. Adoption of strict inclusion criteria may help to gain a result that has more homogenization and repeatability, and the 2018 Movement Disorders Consensus Criteria were adopted to ensure gain a more homogenesis ET group in our study. Second, the small sample studies tend to gain changeful results, and compared to previous studies (not more than 60 ET patients, usually about 40 ET patients), our sample is the largest (101 ET and 105 HCs). Third, the methods of previous studies were the traditional univariate analysis, and the strict multiple comparisons problems prevented taking advantage of all topological metrics information, especially considering the global and nodal topological properties in every single threshold. ML algorithms could automatically capture meaningful information from high-dimensional data to build a stable classification model based on the individual level (Lei et al., 2020), and these merits give a way to use all topological metrics in our study. Finally, the results showed that the metrics under a single threshold also have significant discriminative ability. It is shown that research on a single threshold is meaningful, which is also an extension and refinement of existing research. In conclusion, our results further support GT as a promising and powerful method for identifying underlying brain network topological pathogenesis mechanisms in ET.

In our study, the significant discrimination features were mainly located in the cerebellar-motor network, including the cerebellum, precentral gyrus, SMA, and right pre-SMA. Growing evidence from histopathology, neuroimaging, neurobiology, and electrophysiological have pointed out that the cerebellar-motor cortices network played a vital role in tremor genesis and propagation (Fang et al., 2015, 2016; Louis, 2018; Lan et al., 2021). Meanwhile, using Rs-fMRI analysis methods, studies revealed that changes in ALFF, ReHo, seed-based functional connectivity, and brain global functional connectivity in the cerebellar-motor cortices pathways were associated with tremor in ET patients (Fang et al., 2013; Buijink et al., 2015; Zhang et al., 2022). Again, using GT based on traditional mass univariate analyses also explored that the changes of nodal topological properties in the classical tremor network were involved in ET patients (Benito-León et al., 2019b; Li et al., 2021). Therefore, we believed that our results were consistent with the above-mentioned classical tremor network theory in ET, and further enrich this tremor network theory that the nodal topological properties also served as the significant discrimination features to identify ET from HCs.

Furthermore, the significant discrimination features were also located in cerebellar-non-motor networks, such as the nodal clustering coefficient, degree centrality, and nodal local efficiency in the anterior cingulate cortex, left post occipital, and right occipital. It seems difficult for us to understand that the significant discrimination features were extended out of the classical tremor network and involved in the cerebellar-non-motor pathway. There are several possible explanations for the above aspects: First, most researchers accepted that ET was a cerebellum-caused disease (Louis, 2018). The functional heterogeneity of the cerebellum gives a reasonable interpretation that the dysfunction is not confined to cerebellar-motor circuits but also extended to cerebellar-non-motor cortical pathway (Louis and Faust, 2020; Mavroudis et al., 2022). Second, strict inclusion criteria without gross cognitive impairment, depression, and anxiety were adopted to gain a highly homogeneous ET cohort in our study. However, these strict inclusion criteria could not remove the development of the above non-motor symptoms in the future, and even get rid of a compensatory state to prevent the development of these non-motor symptoms.

And then, in our study, we found that there was no difference in head movement parameters (Friston 24 head motion parameters and FD_power) between the ET patients and HCs. This seems very strange for a group of patients with a movement disorder and we speculated that the following reasons may be a reasonable explanation: Firstly, almost all researchers accept that ET is a heterogeneous disease with a lot of motor and non-motor symptoms (Benito-León et al., 2019b; Li et al., 2021), and besides with tremor in upper limbs also with a tremor in head, jaw, and legs, and tremor in the head seems to cause excessive head movement in ET patients. However, the 2018 Movement Disorders Consensus Criteria redefined that ET is a movement syndrome characterized by isolated tremor of bilateral upper limbs for at least 3 years, and isolated focal tremor in other anatomical distributions (e.g., head, jaw, and legs) did not meet the diagnosis of ET (Bhatia et al., 2018), so the patients with an isolated focal tremor in the head were not included in this study. Secondly, tremors in the upper limbs may cause excessive head movement in ET patients like other movement disorders such as Parkinson’s disease. However, the tremor in Parkinson’s disease is a resting tremor, which is different from the tremor in ET, and the tremor of ET is characterized by action and postural tremor. Meanwhile, the patients are lying on the examining table without action and postural changes during MRI scanning. Therefore, our results further strengthen the tremor theory of ET that ET is a movement disorder disease characterized by action and postural tremor rather than resting tremor in Parkinson’s disease.

Finally, although the current study has some strengths, several limitations need to be acknowledged. First, all subject data were collected in the First Affiliated Hospital of Chongqing Medical University (single-center). The generalization and robustness of the proposed models need to be further verified on the larger and multicenter samples in future studies. Secondly, studies showed that the combination of multimodal neuroimage data could significantly improve the classification performance and provide more information from different views than a single model. In the future study, we hope to classify and explore the pathological mechanisms of ET by combining multimodal neuroimage data, such as FC, ReHo, ALFF, or structural MRI. Finally, due to the lack of objective pathophysiological markers, the diagnosis of ET mainly depends on clinical symptoms and nervous system examination, and misdiagnosis is very common. Therefore, in our study, all patients with ET already had annual follow-ups for more than 3 years to minimize the risk of misdiagnosis.

## Conclusion

Using multiple ML algorithms combined with brain network topological metrics to identify ET from HCs achieved good classification performance. The global and nodal topological metrics served as significant discriminative features, and these features were mostly located in the classical tremor network and cerebello-non-motor networks. These findings help to not only understand the brain network topological pathogenesis mechanisms in ET patients but also establish the potential non-invasive diagnostic image markers.

## Data availability statement

The raw data supporting the conclusions of this article will be made available by the authors, without undue reservation.

## Ethics statement

The studies involving human participants were reviewed and approved by the Ethics Committee of The First Affiliated Hospital of Chongqing Medical University (Chongqing, China). The patients/participants provided their written informed consent to participate in this study.

## Author contributions

QL: conceptualization, investigation, data curation, formal analysis, methodology, software, visualization, writing—original draft, and writing—review and editing. LT: investigation, data curation, formal analysis, methodology, software, supervision, validation, visualization, writing—original draft, and writing—review and editing. PX, HG, and BX: investigation, data curation, formal analysis, methodology, software, and writing—review and editing. XuZ and XiZ: data curation, formal analysis, software, and writing—review and editing. HC, HW, WH, and FL: supervision, validation, and writing—review and editing. OC, JL, YM, and ZX: data curation, supervision, and writing—review and editing. WF: conceptualization, funding acquisition, project administration, supervision, validation, and writing—review and editing. All authors contributed to the article and approved the submitted version.

## Footnotes

1
http://rfmri.org/DPARSF
