# Supplementary material for: Combined brain network topological metrics with machine learning algorithms to identify essential tremor
Source: Front Neurosci. 2022 Nov 2;16:1035153. doi: 10.3389/fnins.2022.1035153 (PMC9667093; doi:10.3389/fnins.2022.1035153)
Supplement: Supplementary file 1 [file Data_Sheet_1.docx]

Supplementary Material

Supplementary text 1: image quality and head motion control criteria

Systematic tactics were used to deal with the major obstacle of the intrinsic BOLD signal when we analyzed the RS-fMRI data, including non-neuronal physiological processes and head motion. First, the subjects who had evidence of vascular or apparent structural brain defects on T2- or T1-weighted images, or whose T1-weighted images and the T2-weighted FLAIR images existed with obvious gross brain structure or signals abnormalities were discarded by visual inspection, and no participants were removed. Second, the Friston 24 head motion parameters were regressed out, including six head-motion parameters (three translational and three rotational), six head-motion parameters one time point before, and the 12 corresponding squared items. Third, we regressed nuisance signals such as white matter (WM), cerebrospinal fluid (CSF), and also global signals. Fourth, we also deal with the volume-to-volume head motion, also called framewise displacement (FDs). Using mean FD estimates of power > 0.2mm as a threshold (not FD_Jenkinson_), the maximal scrubbings volumes were calculated in our study, and two sample t-test was conducted to explore whether these head parameters exist a significant difference between the two groups. The results showed that the maximal scrubbing volumes were 27 volumes (27/230 = 11.73%) in our study. There was no significant difference in scrubbing volumes and the mean FD estimates of power between the two groups (scrubbing volumes:15.21 ± 7.95, 16.61 ± 9.12, T = -1.17, *P* = 0.2437; mean FD_power: 0.10 ± 0.06, 0.01 ± 0.06, *T* = 0.59, *P* = 0.5562).

Supplementary Table 1. Top 13 topological features with the maximum discriminative ability (occurrence frequency greater than 80%) selected by the Mann-Whitney U test and LASSO.

| Thresholds | Topological metrics | The brain areas number | Corresponding brain regions | Subnetwork | Coefficient | Frequency |
| --- | --- | --- | --- | --- | --- | --- |
| 0.20 | Eglobal |  |  |  | 0.1825 | 99 |
| 0.05-0.50 | Degree centrality | 101 | right precentral gyrus | Sensorimotor | 0.0925 | 99 |
| 0.05-0.50 | Degree centrality | 135 | left post occipital | Occipital | 0.0839 | 100 |
| 0.05-0.50 | Nodal clustering coefficient | 58 | left ACC | Cingulo-opercular | 0.0563 | 99 |
| 0.05-0.50 | Degree centrality | 97 | left precentral gyrus | Sensorimotor | 0.0323 | 82 |
| 0.05-0.50 | Degree centrality | 93 | SMA | Sensorimotor | 0.0294 | 84 |
| 0.05-0.50 | Nodal clustering coefficient | 8 | right ACC | DMN | 0.0275 | 89 |
| 0.05-0.50 | Nodal local efficiency | 127 | right occipital | Occipital | 0.0253 | 87 |
| 0.05-0.50 | Degree centrality | 91 | right pre-SMA | Sensorimotor | 0.0231 | 81 |
| 0.05-0.50 | Degree centrality | 150 | left med cerebellum | Cerebellum | -0.0283 | 83 |
| 0.05-0.50 | Nodal efficiency | 96 | left mid insula | Sensorimotor | -0.0323 | 80 |
| 0.35 | Degree centrality | 147 | left med cerebellum | Cerebellum | -0.0330 | 95 |
| 0.05-0.50 | Nodal local efficiency | 150 | left med cerebellum | Cerebellum | -0.0626 | 95 |

LASSO: the least absolute shrinkage and selection operator. *P* values of the top 13 features were less than 0.01. Eglobal: global efficiency; med cerebellum: medial cerebellum; mid insula: middle insula; ACC: anterior cingulate cortex; SMA: supplementary motor area.

Supplementary Table 2. The topological metrics of the top 13 features in the ET group *vs* HCs.

| Topological metrics | Thresholds | ET > HCs | Thresholds | ET < HCs |
| --- | --- | --- | --- | --- |
| Eglobal  Nodal clustering coefficient  Degree centrality  Nodal local efficiency | 0.20  0.05-0.50  0.05-0.50  0.05-0.50  0.05-0.50  0.05-0.50  0.05-0.50  0.05-0.50  0.05-0.50 | Eglobal  right ACC  left ACC  left post occipital  SMA  right precentral gyrus  left precentral gyrus  right pre-SMA  right occipital | 0.05-0.50  0.35  0.05-0.50 | left med cerebellum  left med cerebellum  left med cerebellum |
| Nodal efficiency |  |  | 0.05-0.50 | left mid insula |

ET: essential tremor, HCs: healthy controls. Eglobal: global efficiency; med cerebellum: medial cerebellum; mid insula: middle insula; ACC: anterior cingulate cortex; SMA: supplementary motor area.

Table S3. Classification performance of multiple classifiers in the training dataset.

| model | Training dataset | | | | |
| --- | --- | --- | --- | --- | --- |
|  | mACC (%) | mb-ACC (%) | mSEN (%) | mSPE (%) | mAUC |
| SVM | 99.05±1.27 | 99.05±1.27 | 99.14±1.38 | 98.96±6.40 | 0.994 |
| LR | 100.00±0.00 | 100.00±0.00 | 100.00±0.00 | 100.00±0.00 | 0.995 |
| RF | 100.00±0.00 | 100.00±0.00 | 100.00±0.00 | 100.00±0.00 | 0.995 |
| GussionNB | 93.23±1.70 | 93.22±1.70 | 92.89±2.76 | 93.56±2.22 | 0.975 |

Data are shown as means ± standard deviations.

mACC, mean accuracy; mb-ACC: mean balanced accuracy; mSEN, mean sensitivity; mSPE, mean specificity; mAUC, mean area under receive operator curve. SVM, support vector machine; RF, random forest; LR, logistic regression; GussianNB, guassian naïve bayes.
